# Dermatologic laser-induced ocular and periocular complications: a review

**DOI:** 10.1186/s12886-023-03159-x

**Published:** 2023-10-19

**Authors:** Karolina Bonińska

**Affiliations:** Miejskie Centrum Medyczne Jonscher, ul. Milionowa 14, 93-113 Łódź, Poland

**Keywords:** Periocular laser complications, Ocular laser complicatons, Dermatologic laser complications, Cosmetic laser complications

## Abstract

This study aimed to discuss severe ocular and periocular complications after dermatological laser treatment. This literature review evaluated published journal articles (clinical trials or scientific reviews) extracted from electronic databases (MEDLINE and PubMed) and the reference lists of related articles. Individual eyeball structures, such as chromophores, were found to interact with laser radiation. The type of damage was found to be influenced by the wavelength emitted by the laser-active medium. Moreover, in the absence of proper protection of the eyeballs, the structures that are crucial for vision can be damaged. In conclusion, laser treatment can also cause serious and irreversible complications. Appropriate patient selection, intraoperative techniques, early identification, and interventions for adverse postoperative effects are crucial to avoid major complications and even blindness.

## Introduction

Lasers are popular tools in numerous branches of medicine owing to the versatile benefits of their applications. The active center of the laser determines the light length that the laser will emit and the structures it will act on. Depending on the wavelength, the energy of the laser pulse in tissues is absorbed by water, melanin, or hemoglobin. However, different effects are observed depending on the power density of the laser pulse (energy) delivered to the tissues [1].

The most common applications in aesthetic medicine or cosmetology involve lasers that produce photochemical effects for tissue biostimulation, as well as photothermal (non-ablative lasers) and photoablative lasers that cause rapid tissue vaporization [1].

To achieve optimal therapy results, it is necessary to correctly select the patient, choose the right type of laser, and familiarize the patient with the modes of operation. This minimizes the risk of possible complications.

This study aimed to discuss significant ophthalmic complications arising from the inappropriate use of laser energy, which have serious implications for visual organs. To the best of my knowledge, this is the first study to describe the pathomechanism of the resulting damage due to the use of laser and therapeutic options. Complications are described, from the least serious to the most serious ones.

## Literature search

This literature review included journal articles (clinical trials and scientific reviews) published up to March 2023. Studies were identified by searching electronic databases (MEDLINE and PubMed) and the reference lists of related articles. The following keywords were used in various combinations to extract the relevant studies: “periocular laser complications,” “ocular laser complicatons”, dermatologic laser complications,” and “cosmetic laser complications.” In addition, the references cited in the identified articles were reviewed to identify additional reports.

### Lower eyelid turned outwards (ectropion)

Ectropion is a laser resurfacing complication of the periocular region resulting from ablation methods, most often using a CO_2_ laser (10,600 nm). This can result from scarring, excessive energy flow, or density [2, 3]. Ectropion leads to chronic lacrimation, eyelid and eyeball conjunctivitis, and permanent corneal damage. It is necessary to use eyelid massage and Pulsed Dye Laser treatment to prevent this from happening. Eyelidplasty is necessary for patients with chronic ectropion [4].

Patients with involutional stretching of the lateral eyelid ligament and a history of lower eyelid surgery are at risk of ectropion [3–4].

Preoperatively, eyelid tension should be assessed using the snap-back test. When the lower eyelid is pulled back, it quickly returns to its original location. The longer the duration, the greater the stretch. Furthermore, it is advisable to use a lower energy density and intraoperatively evaluate the tissue response to the laser [4].

### Cataract with iris atrophy

The literature describes a case of cataract development with iris atrophy in a patient with eyebrow epilation [5]. The procedure was performed 2–5 mm below the eyebrow using a diode laser (800 nm). Eye protection was not provided. During the procedure, the patient reported pain in the left eye with decreased vision and increased sensitivity to light. At a follow-up visit six weeks later, the iris was noted to have a defect in its superior part, and there was a nuclear cataract. The patient had a visual acuity of 20/30. The patient did not require any further treatment.

Epilation lasers produce a photothermolytic effect with a wavelength of 600–1100 nm, which is absorbed by melanin [6]. The penetration depth through the thin eyelid skin and naturally occurring Bell’s reflex, or the upward positioning of the eyeball when the eyelids are closed, increase the risk of eye damage. Eye protection is recommended for all laser epilation procedures [5].

### Uveitis with corneal damage

Another complication of eyebrow epilation is anterior segment uveitis after the use of an alexandrite laser (755 nm) [7–10]. Eye protection was not provided. The reported complaints included eye pain, photophobia, and cloudy vision. Ophthalmologic examination revealed conjunctivitis, deep injection, inflammatory cells in the anterior chamber, iris inflammation, iris thinning, and a lazy-pupil response to light. Therefore, topical treatment with steroid drops and cycloplegics was necessary. Intraocular pressure remained normal. No abnormalities were observed in the posterior segment of the eyeball, and the visual acuity was high (20/20).

The inflammation in the described cases resolved after treatment. The damage to the iris manifested as thinning and rounding of the pupil and was permanent. However, it did not affect the final visual acuity.

### Retinal burns with vitreous hemorrhage

This rare complication occurs after the use of a Q-switched Nd:YAG (1064 nm) laser in the absence of eye protection [11]. Immediately after exposure, the patient reported a flash in the eye, with the subsequent appearance of floaters. The pulse duration and laser energy were not assessed. Visual acuity, intraocular pressure, and the anterior segment of the eye were normal. Fundus examination revealed a lesion in the inferior part of the retina with a hemorrhage into the vitreous body. The macular area was normal. Oral steroids and vitamins were administered. The number of floaters decreased after a few days. There was a peripheral retinal edema noted in addition to the hemorrhage. Subsequent follow-ups were not reported.

The authors noted the complex nature of resulting retinal damage. Anti-inflammatory and oxidative stress-reducing treatments, such as steroids, vitamins, and supplements, are expected to play an important role [11].

### Maculopathy

Macular damage occurs after alexandrite laser epilation (755 nm) in the absence of protective shielding [10]. The visual acuity after the procedure was 20/200. Macular SD-OCT nad fluorescein angiography confirmed the presence of a subretinal hemorrhage with neovascular membrane development and fluid in the neurosensory retina 20 days after procedure. An anti-vascular endothelial growth factor agent was injected into the vitreous chamber to reverse the resulting lesions. After 1 month, there was an improvement in visual acuity (20/20). with complete improvement in morphological imaging.

Neovascular membrane development is a disease that reduces central vision. Rapid treatment prevents permanent and irreversible damage to the macula.

### Corneal ulceration with cataract development

This serious complication occurred in the patient as a result of periorbital skin resurfacing with a CO_2_ laser [12]. Protective steel Cox II shields were used during inflammatory treatment, includinglaser treatment. Shortly after the procedure, the patient reported visual deterioration. Ophthalmological examination revealed reduced visual acuity (VA) in both eyes (RE = 0.6; LE = 0.2). In the right eye, corneal epithelial haze was observed. In the left eye, superficial and deep corneal haze, erosion, stromal edema, limbal ischemia, and pupil dilation were observed. The patient received dexamethasone-tobramycin ointment, 1% cyclopentolate drops, serum and cyclosporine drops, vitamin C tablets, and oral doxycycline. At the last follow-up visit after 2 years, the visual acuity in the right eye was 1.25, and 2/300 in the left eye. No abnormalities are observed in the right eye. The cataract was removed from the left eye. In addition, scarring with vascularization and corneal thinning has been reported [12].

This case, unlike those described above, resulted from the use of eyeball protection. Accidental metal shield targeting leads to overheating of the anterior eyeball The laser effect is enhanced by the lacrimal film. The calculated temperature can exceed 70 °C [12].

## Discussion

The individual structures of the eyeball act as chromophores for the laser beams. Tissue damage sites depend on the length of the light emitted by the active medium of the laser [13].

For the retina, the hazardous radiation lengths were within 400–1400 nm. Thus, they cover the range of visible light (400–780 nm) and near-infrared (780–1400 nm) light. Since the retina does not contain pain receptors and does not have the ability to perceive wavelengths above 780 nm, exposure to near-infrared light can go unnoticed until serious damage occurs, including impaired night vision, color vision, and total blindness. Accidental exposure to an Nd:YAG laser beam is the most commonly recorded cause of retinal damage [13–15].

Mid-infrared (1400–3000 nm) and far-infrared (3000–10,600 nm) wavelengths are mainly absorbed by water. The structures containing most of these are the cornea and lens. Possible damage includes corneal surface burns and translucency disorders [16, 17]. These can be induced using CO_2_ and Er:YAG lasers [16, 18]. Most superficial injuries heal within a few days, without permanent sequelae. Deep-penetrating lasers operating in the mid-infrared region can cause the denaturation of proteins present in the lens of the eye, leading to opacity or cataracts. In addition, the near-UV range (315–400 nm), which passes through the cornea and becomes concentrated in the eye lens, poses a threat to cataract formation [13].

The light ranges that can damage both the retina and lens simultaneously include the transition from UV to visible light, which is approximately 400 nm, and the near-infrared to the mid-infrared range, which is approximately 1300 nm [13, 16]. Currently, there are no devices in the aesthetic dermatology market operating in the 400 nm range. However, a 1320 nm Nd:YAG laser is used for non-ablative rejuvenation treatments [13, 19].

Working with laser energy may damage not only the patient’s eyes but also the eyes of the person performing the procedure. Complications can occur owing to the direct impact of the laser beam, reflection, or diffusion. Laser eye protection is necessary when using Class 3 B and Class 4 lasers [13, 20]. No protection was provided during the procedures for most complications described herein [5, 7–11]. Commercially available shields include protective goggles, face shields, glass with special filters, and reflective coating [20–22]. The person performing the procedure was responsible for selecting the correct laser and protective procedures. Care should be taken to ensure proper fittings and disinfection.

As demonstrated in the last case of corneal ulceration, eye protection does not prevent all possible complications when showing carelessness in directing the laser beam [12]. Additional protection is provided by dry cotton rounds inserted under the shields, which fit tightly against the orbital bone, eliminating the risk of overheating the anterior structures of the eyeball and radiation leakage with ill-fitting shields [13].

This study lacks a discussion of the complications resulting from the selected dermatological lasers, posing a disadvantage. This is because it represents a subjective selection of complications relevant to the ophthalmologist’s perspective, with emphasis on the damage caused. The improvement of laser techniques and the implementation of new laser types will become the basis of numerous studies that describes the multidimensional effects of the use lasers and possible potential complications.

## Conclusions

Any deterioration in vision during or after laser therapy should be consulted with an ophthalmologist as soon as possible. This can prevent serious complications, up to and including blindness.

## Data Availability

Data is contained within the article.
